# Navigating the management of open apex: A review

**DOI:** 10.6026/973206300212299

**Published:** 2025-08-31

**Authors:** Ketaki Rajguru, Pranjali Dutt, Prerna Priya, Deepti Singh, Chanda Dhakad, Nikhil Sathawane, Mahesh D.R., Pratik Surana

**Affiliations:** 1 Department of Conservative Dentistry and Endodontics, Tatyasaheb Kore Dental College and Research Centre, Nave Pargaon, Maharashtra, India; 2 Department of Dentistry, Mahamaya Rajkiya Allopathic Medical College, Ambedkarnagar, Uttar Pradesh, India; 3 Department of Dentistry, AIIMS Rishikesh, Uttarakhand, India; 4 Department of Conservative Dentistry and Endodontics, Faculty of Dental Sciences, IMS BHU, Varanasi -221005, Uttar Pradesh, India; 5 Department of Conservative Dentistry and Endodontics, Swargiya Dadasaheb Kalmegh Smruti Dental College and Hospital, Nagpur, Maharashtra, India; 6 Department of Oral Medicine and Radiology, Dayananda Sagar College of Dental Sciences, Shavige Malleshwara Hills KS Layout, Bangalore, Karnataka, India; 7 Department of Pedodontics and Preventive Dentistry, Maitri College of Dentistry and Research Centre, Durg, Chhattisgarh, India

**Keywords:** Open apex, apexification, revascularization

## Abstract

The management of open apex teeth, highlighting traditional apexification and contemporary revascularization techniques is of interest. Apexification, a long-established approach, promotes apical barrier formation, while revascularization offers the potential for continued root development, especially in younger patients. The selection of treatment should be tailored to individual cases, prioritizing the preservation of tooth integrity and optimal outcomes.

## Background:

In endodontics, the term "open apex" describes a scenario where the root tip has not developed fully, which brings its own unique set of challenges when diagnosing and treating dental issues. In clinical practice, we often see teeth with open apices, especially in situations involving trauma, developmental issues, or young permanent teeth. The distinct anatomical features of these teeth, characterized by an unclosed root tip, complicate traditional endodontic treatments, requiring us to adopt innovative and evolving strategies. This open apex condition occurs when the normal maturation of the root is interrupted, preventing the formation of a complete apical foramen [1, 2]. When dealing with a permanent tooth that has short roots and needs endodontic treatment, one possible approach is apexification. This technique works to create a calcified barrier at the tip of a root that hasn't fully formed, particularly when the pulp is found to be necrotic [3]. Apexification often requires several monthly visits to place calcium hydroxide (Ca(OH)_2_) in the root canal, eliminating infection and promoting calcification for apical closure [4]. After a few months, X-rays should show thicker canal walls and a rounded apex, allowing for sealing with gutta-percha and a sealing agent. However, Ca(OH)_2_ may weaken dentin, increasing fracture risk [5, 6]. The traditional use of Ca(OH)_2_ for apexification is being gradually replaced by mineral trioxide aggregate (MTA), which can be applied in one step and serves as an apical plug or canal filling after disinfection [7]. Therefore, it is of interest to describe various techniques for the management of open apex.

## Etiology of open apex:

The etiology of open apex primarily includes trauma, which is a leading cause of this condition in teeth. Trauma can significantly disrupt pulpal microcirculation, leading to pulpal necrosis and the cessation of root formation. Additionally, thermal and chemical injuries can adversely affect the pulpal tissue, resulting in incomplete root development. Iatrogenic factors, such as improper management of working length during treatment, can also cause root end enlargement, whether through manual or rotary instruments. Other contributing factors include dental anomalies like dens evaginatus and dens invaginatus [8].

## Classification of open apex:

Open apices can be classified into two main types: blunderbuss and non-blunderbuss apices [9].

## Blunderbuss apex:

This type features divergent walls with a funnel-shaped flare at the apex.

## Non-Blunderbuss apex:

In this case, the canal walls can be parallel or slightly convergent, resulting in a broader or more convergent apex.

## Diagnosis of open apex:

To assess permanent teeth with immature roots, clinicians perform various tests, including electric and thermal evaluations, which can yield inconsistent results. Doppler flowmetry measures blood flow in injured teeth, while a pulse oximeter checks pulp vitality by monitoring oxygenation [10]. A thorough dental history is vital for understanding pain symptoms, such as duration and aggravating or relieving factors. Clinical exams should also look for signs like swelling, discoloration, decay, mobility, and periodontal probing. Interpreting radiographic images can be tricky, especially in distinguishing healthy pulp from necrotic ones; comparing findings with the apex of a healthy opposing tooth can help clarify this [9]. Cone-beam computed tomography (CBCT), also known as digital volume tomography, overcomes the shortcomings of traditional two-dimensional X-rays by delivering accurate measurements of dentinal walls and periapical lesions. By enabling early diagnosis and intervention with these cutting-edge imaging methods, we can significantly improve pulp preservation strategies, fostering an ideal environment for on-going dentin growth and root development [11].

## Apexification:

Apexification is a procedure designed to facilitate the formation of an apical barrier, effectively sealing the open apex of an immature tooth with a nonvital pulp, allowing for containment of filling materials within the root canal space. As part of this process, immature teeth are typically disinfected using irrigants such as NaOCl, chlorhexidine, EDTA and iodine-potassium iodide. Following disinfection, the canal is filled with calcium hydroxide paste to promote further disinfection and stimulate the formation of an apical calcific barrier. Ca(OH)_2_ exhibits antimicrobial properties through the release of hydroxyl ions, which can damage bacterial cellular components [12]. Filling the root canal typically occurs once the apical calcific barrier has formed. In the absence of this barrier, there is no resistance for the traditional gutta-percha filling material to be properly condensed against. In addition to its role as an effective disinfectant, early research has indicated that calcium hydroxide may possess osteo-inductive properties [13]. The high pH of calcium hydroxide is believed to play a role in inducing hard tissue formation [14]. However, the time required for apical barrier formation using Ca(OH)_2_ can be considerable, often taking as long as 20 months. Factors such as the patient's age and the presence of symptoms or periradicular radiolucencies may also influence the duration needed for barrier formation. Refreshing the Ca(OH)_2_ paste typically occurs every three months [12]. Several shortcomings associated with Ca(OH)_2_ apexification can be noted: (i) the lengthy treatment duration; (ii) the necessity for multiple visits, which places significant demands on both patients and caregivers, along with increased clinical costs; and (iii) a higher risk of tooth fractures when using Ca(OH)_2_ as a long-term root canal dressing. These drawbacks have led to the adoption of mineral trioxide aggregate as an alternative, allowing for filling the apical end without requiring the formation of a calcific barrier [15].

## Single sitting apexification:

Single sitting apexification is an innovative endodontic technique that utilizes mineral trioxide aggregate to close the open apex of an immature tooth in a single appointment. This approach offers several advantages over traditional methods that employ calcium hydroxide, including reduced treatment duration, as it eliminates the need for multiple visits and prolonged treatment periods. Consequently, it enhances patient compliance by minimizing the risk of lost follow-ups and simplifying the overall process [16]. MTA provides superior sealing properties compared to calcium hydroxide, effectively preventing bacterial infiltration and ensuring a stable coronal seal. Additionally, MTA is known for its excellent biocompatibility, promoting healing while reducing the likelihood of adverse reactions in surrounding tissues. As a bioactive material, MTA also encourages the formation of new mineralized tissues, supporting the natural healing process of the tooth. In this procedure, the canal is thoroughly disinfected before MTA is directly placed to fill the apical region, creating an apical barrier in a single visit. This technique is especially beneficial for young patients and those with limited ability to attend multiple appointments, leading to favorable treatment outcomes and increased patient satisfaction. Overall, single sitting apexification represents a valuable advancement in endodontics for effectively and efficiently managing open apex cases [17, 18]. Recently, Biodentine, calcium silicate-based cement, has emerged as a new option for apical barrier formation. This innovative bioactive dentin replacement cement is formulated from a powder that contains calcium carbonate, zirconium oxide, tricalcium silicate, dicalcium silicate, and calcium hydroxide. One of the key advantages of Biodentine is its ease of preparation and handling, making it user-friendly for clinicians. Additionally, it offers a significantly shorter setting time compared to other silicate-based cements; while mineral trioxide aggregate typically takes about 2 hours and 45 minutes to set, Biodentine sets in just 12 minutes. This rapid setting property allows for quicker treatment procedures and enhances the overall efficiency of apexification therapy [18].

## Revascularization:

While the standardized clinical approach for apexification has been widely practiced, some clinicians inevitably modify their treatment procedures based on their clinical judgment and individual case requirements. Various practitioners have reported using alternative approaches, and three specific methods have garnered significant interest from the endodontic community. These innovative strategies reflect clinical adaptability and the pursuit of improved outcomes in managing open apex cases, showcasing the evolving nature of endodontic practices and the willingness of clinicians to explore new techniques for enhanced patient care. Since the dawn of the 21st century, we've gained the ability to create a biological barrier through the potential of stem cell differentiation, a process often called "revascularization." Unlike traditional apexification, this cutting-edge technique enables root development to proceed with the patient's own stem cells. Nevertheless, this treatment remains under-researched, raising questions and debates about the origin and characteristics of the tissue that forms within the canal [19]. Shimizu and colleagues (2012) found that pulp-like tissue can regenerate post-revascularization in an immature permanent tooth affected by irreversible pulpitis. Remarkably, both the apical papilla and Hertwig's epithelial root sheath stayed intact in the presence of pulpitis, showing no indications of apical periodontitis [20]. This area of research highlights the potential for regenerative endodontic techniques to transform the management of teeth with nonvital pulps. Some authors have hypothesized the presence of vital pulp cells that may serve as the origin for the neoformed tissue in revascularization cases [19].

## Revascularization procedure:

The elimination of dead microorganisms and pulp tissue from the root canal is vital for the success of the treatment process. Hence, clinicians have a broad range of disinfecting agents at their disposal, with sodium hypochlorite being the most widely utilized. In addition to irrigation solutions, intracanal medications have shown effectiveness in combating infections [19]. In addition to irrigation solutions, intracanal medications have shown effectiveness in treating infections. Hoshino (1996) introduced a tri-antibiotic paste consisting of metronidazole, ciprofloxacin, and minocycline which can effectively eliminate Gram-positive, negative and anaerobic bacteria. However, high concentrations of this paste may negatively impact stem cells [21]. Positive outcomes of revascularization are heavily dependent on the formation of a blood clot. This process involves the use of endodontic instruments, such as K or H files, which extend beyond the apical region of a disinfected tooth. If there is insufficient bleeding, root development will be hindered. Once the blood clot stabilizes, a scaffold is introduced. These scaffolding materials play a crucial role in ensuring the treatment's success by maintaining the function and vitality of the regenerated tissue [22]. In addition to root canal disinfection and the implementation of an appropriate scaffold, the quality of the coronal restoration is vital for the success of revascularization treatment. Achieving a bacterial-tight coronal seal is essential, and this can be accomplished using materials such as composite resin, MTA, glass ionomer, or various combinations of these materials [23].

## Discussion:

According to Nadgouda *et al.* the management of open apex cases in endodontics presents unique challenges, particularly in achieving root maturation and preventing infection. Among the various techniques to treat teeth with an open apex, apexification and revascularization are two prominent approaches, each with its own indications and outcomes [24]. To address the challenges associated with open apex cases, various therapeutic solutions have been proposed, with apexification being the most established technique. The American Association of Endodontists defines apexification as "a method to induce a calcified barrier in a root with an open apex or to facilitate the continued apical development of an incomplete root in teeth with necrotic pulp [19]." Calcium hydroxide has been the preferred material for this procedure for several decades, first introduced by Hermann in 1920. Its use in apexification gained prominence in the 1960s through the work of Kaiser and Frank, who successfully demonstrated its ability to promote the formation of a hard tissue barrier in under developed teeth with necrotic pulps [25]. Calcium hydroxide's effectiveness in apexification can be attributed to its diverse biological effects, which include antimicrobial properties and the ability to stimulate tissue regeneration. However, the duration required for apexification though Ca(OH)_2_ can vary significantly, ranging from 3-24 months to obtain complete root closure. This variability is influenced by factors such as the individual patient's healing response, the initial tooth condition, and the presence of infection. Despite these timeframes, the long history of successful outcomes with calcium hydroxide in apexification has solidified its role as a cornerstone in the management of open apex cases [26].

Mineral Trioxide Aggregate exhibits a high alkalinity (12.5 pH), which is comparable to that of calcium hydroxide, which has a pH of 12. This similarity in pH contributes to the activation of alkaline phosphatase and enhances antibacterial properties. The composition of MTA, which includes several calcium salts, further elevates calcium concentration, boosting the function of calcium-dependent pyrophosphatase. This process aids in achieving asepsis in lesions while initiating bone healing [27]. Clinical studies have demonstrated that apexification using MTA presents a feasible alternative for achieving root completion in teeth with an open apex. Mente *et al.* conducted a study with on apexification, involving 252 cases and a 10year follow. The results show that the success rates for teeth with open apices using apical plugs with MTA is an effective treatment option [28]. Revascularization, alternatively, is a relatively newer technique that aims to restore the natural regenerative capacity of the pulp. This approach involves inducing bleeding into the root canal space to promote the formation of a blood clot, which then serves as a scaffold for stem cells to migrate, proliferate, and form new vascularized pulp tissue [19]. Belli *et al.* (2018) found that the revascularization technique was biomechanically superior to apexification [29]. Cehreli *et al.* observed root development and wall thickening in six immature permanent teeth over a 10-month follow-up [30]. Similarly, Reynolds *et al.* reported significant lesion reduction (60% of treated teeth) in a 24-month clinical and radiological follow-up of five immature teeth with apical lesions [31]. One of the significant advantages of revascularization is that it not only closes the apex but also promotes the continued growth and maturation of the root structure, potentially leading to greater root thickness and strength [30]. When compare apexification and revascularization, the choice of approach depends on several clinical considerations, including the age of the patient, the condition of the tooth, and the presence of infection. Apexification may be more suitable for older patients or instances where the tooth is unlikely to respond to revascularization. Conversely, revascularization is often preferred for younger patients, as it provides the opportunity for continued root development. Both techniques require careful assessment and planning to ensure optimal outcomes. A thorough understanding of the biological processes involved in healing and regeneration will guide clinicians in selecting the most appropriate treatment strategy for managing open apex cases.

## Conclusion:

Both apexification and revascularization provide important treatment options for open apex teeth. While apexification has been the traditional method, advancements in revascularization can lead to better outcomes, particularly for younger patients. The choice between the two techniques should depend on the clinical situation, focusing on preserving tooth vitality and supporting proper development.
